# Features Predicting Weight Loss in Overweight or Obese Participants in a Web-Based Intervention: Randomized Trial

**DOI:** 10.2196/jmir.2156

**Published:** 2012-12-12

**Authors:** Emily Brindal, Jill Freyne, Ian Saunders, Shlomo Berkovsky, Greg Smith, Manny Noakes

**Affiliations:** ^1^CSIROFood & Nutritional SciencesAdelaideAustralia; ^2^CSIROICT CentreMarsfieldAustralia; ^3^CSIROMathematics, Informatics and StatisticsWaite CampusAustralia

**Keywords:** Internet, obesity, dietetics

## Abstract

**Background:**

Obesity remains a serious issue in many countries. Web-based programs offer good potential for delivery of weight loss programs. Yet, many Internet-delivered weight loss studies include support from medical or nutritional experts, and relatively little is known about purely web-based weight loss programs.

**Objective:**

To determine whether supportive features and personalization in a 12-week web-based lifestyle intervention with no in-person professional contact affect retention and weight loss.

**Methods:**

We assessed the effect of different features of a web-based weight loss intervention using a 12-week repeated-measures randomized parallel design. We developed 7 sites representing 3 functional groups. A national mass media promotion was used to attract overweight/obese Australian adults (based on body mass index [BMI] calculated from self-reported heights and weights). Eligible respondents (n = 8112) were randomly allocated to one of 3 functional groups: information-based (n = 183), supportive (n = 3994), or personalized-supportive (n = 3935). Both supportive sites included tools, such as a weight tracker, meal planner, and social networking platform. The personalized-supportive site included a meal planner that offered recommendations that were personalized using an algorithm based on a user’s preferences for certain foods. Dietary and activity information were constant across sites, based on an existing and tested 12-week weight loss program (the Total Wellbeing Diet). Before and/or after the intervention, participants completed demographic (including self-reported weight), behavioral, and evaluation questionnaires online. Usage of the website and features was objectively recorded. All screening and data collection procedures were performed online with no face-to-face contact.

**Results:**

Across all 3 groups, attrition was high at around 40% in the first week and 20% of the remaining participants each week. Retention was higher for the supportive sites compared to the information-based site only at week 12 (*P* = .01). The average number of days that each site was used varied significantly (*P* = .02) and was higher for the supportive site at 5.96 (SD 11.36) and personalized-supportive site at 5.50 (SD 10.35), relative to the information-based site at 3.43 (SD 4.28). In total, 435 participants provided a valid final weight at the 12-week follow-up. Intention-to-treat analyses (using multiple imputations) revealed that there were no statistically significant differences in weight loss between sites (*P* = .42). On average, participants lost 2.76% (SE 0.32%) of their initial body weight, with 23.7% (SE 3.7%) losing 5% or more of their initial weight. Within supportive conditions, the level of use of the online weight tracker was predictive of weight loss (model estimate = 0.34, *P* < .001). Age (model estimate = 0.04, *P* < .001) and initial BMI (model estimate = -0.03, *P* < .002) were associated with frequency of use of the weight tracker.

**Conclusions:**

Relative to a static control, inclusion of social networking features and personalized meal planning recommendations in a web-based weight loss program did not demonstrate additive effects for user weight loss or retention. These features did, however, increase the average number of days that a user engaged with the system. For users of the supportive websites, greater use of the weight tracker tool was associated with greater weight loss.

## Introduction

Overweight and obesity remain serious concerns for a high proportion of people, with the World Health Organization estimating that 1.5 billion adults were overweight or obese in 2008 [1]. The expanding reach and capability of electronic tools has resulted in increasing interest in eHealth weight loss strategies.

While the evidence surrounding eHealth strategies is evolving, they remain a popular option for delivering health behavior change programs to a widening population. In Australia, the Internet has the potential for wide reach with over 70% of people having access in their home [2]. The potential impact of Internet-delivered programs at a population level is one of its many appeals [3]. Internet-delivered obesity interventions may be more cost-effective than in-person interventions [4,5], with one study indicating that use of a web-based program can reduce actual health care costs [6].

Several reviews of the effectiveness of web-based weight loss interventions have concluded that the evidence is mixed owing, in part, to the diversity of intervention programs evaluated [7-11]. Nevertheless, the American Heart Association [12] recently released a scientific statement suggesting that the Internet could be a promising tool for promoting weight loss. Website usage and self-monitoring seem to be consistently associated with weight loss [10,11].

A point of contention regarding online interventions is their effectiveness in the absence of personal contact. Some reviews suggest that the Internet may provide an effective alternative to traditional face-to-face programs [11], while others question the utility of this approach [13]. Relatively few studies report on web-based weight loss interventions without personal contact. For example, interventions have provided web-based weight maintenance sites after traditional in-person weight loss programs [14,15] or incorporated online components together with face-to-face counseling [16,17]. Those studies that have evaluated the efficacy of pure Internet interventions have largely failed to find additive weight loss benefits of web-based programs relative to control conditions without an Internet component [18,19]. In a recent intervention targeting dietary and physical activity behaviors (not strictly weight loss), Kelders et al [20] also found no differences in behaviors between users who had free access to a website compared to a wait-listed control group. Although these studies have not found additive benefits of Internet delivery relative to usual-care controls, Internet intervention groups often demonstrate a mean weight loss. For example, Gold et al [21] report weight loss of 3.3±5.8 kg over 6 months when participants used the site eDiets.com (with no personal support) but found better weight loss for their comparison website, which included individualized support from a therapist.

One of the issues with purely Internet-delivered interventions is maintaining participant engagement with the sites provided [20,22]. McConnon et al [18] reported 53% of their participants accessed their website. It is possible that enhanced features and social networking tools act to improve engagement with an online intervention, which consequently improves compliance to the weight loss program. A randomized controlled trial comparing the efficacy of enhanced website features at achieving weight loss, while retaining limited contact with participants, indicated that a more intelligent system (ie, one with action plans and self-help advice personalized to individual characteristics) may be beneficial for weight loss relative to basic information presented online [23]. More recently, van Genugten et al [24] compared a structured and interactive weight maintenance website to a static one but found no differences for body mass index (BMI) or weight circumference at 6 months. In the realm of physical activity, Internet interventions have shown promising results for more interactive websites in terms of behavior change [25] or retention [26]. However, not all results have supported additive effects of interactivity for increasing physical activity in web-based interventions [27].

Limited studies have attempted to evaluate the relative advantage of different styles of weight loss websites while also restricting in-person contact with volunteers. Intervention characteristics such as face-to-face contact and individual emails constructed by professionals reduce the real-world translation of web-based interventions and limit the advantages of “direct-to-consumer” [22] programs. The aim of this study was to investigate whether enhanced features and perceived social support through social networking tools in an Internet-only intervention lead to engagement and improved weight loss. We anticipated that higher levels of interactivity, in particular the addition of interactive features including social networking features, as well as personalized planning assistance, would be associated with greater retention and weight loss. Finally, we also aimed to investigate whether particular site features would be associated with higher weight loss.

## Methods

### Study Design

We assessed the effect of different features of a web-based weight loss intervention using a 12-week repeated-measures randomized parallel design. All study components including the intervention, registration and screening processes, randomization, and questionnaires were completed online thereby excluding personal contact with participants within all aspects of the study. This study was approved by the CSIRO Human Research Ethics Committee in December 2010. Due to an administrative oversight, the study was not registered prospectively in a clinical trials registry; the original study protocol is provided in lieu of registration (see Multimedia Appendix 1).

Seven versions of the website were developed (see Multimedia Appendix 2). Many of these sites had the same basic functionality but varied according to different information and communication technology features for the purposes of evaluating human-computer interaction (see [28]). The 7 sites represented 3 functional groups (see Table 1): (1) Information-based: a static non-interactive version of the weight loss program, (2) Supportive: a social, interactive website that provided dietary information as provided in the information-based site, in addition to interactive tools such as real-time dietary compliance visualizations, an interactive meal planner, and social support through a social networking platform (ie, personal profiles, friend networks, blogs, discussion forums, and news feeds), and (3) Personalized-supportive: identical to the supportive version with the addition of a personalized meal planner. This meal planner offered 3 breakfast, lunch, and dinner suggestions personalized to user preferences through a purpose-built algorithm that collated data on user preference ratings of a collection of recipes and previous planning.

**Table 1:** Functionalities of different versions of the websites.

Version	Diet and exercise information	Interactive Planner	Compliance feedback	Social support system	Diet & weight self-monitoring	Personalized planning
Information-based	Y	N	N	N	N	N
Supportive	Y	Y	Y	Y	Y	N
Personalized-supportive	Y	Y	Y	Y	Y	Y

### Participants and Procedure

In July 2010, the Online CSIRO Total Wellbeing Diet (TWD) [29] study was promoted in national mass media using the tag line, “CSIRO are looking for participation in an online diet study”. Interested participants were instructed to register online. To be eligible to participate in the trial, registrants needed to be adults (18 years and over) with a BMI >25kg/m^2^ (calculated using self-reported heights and weights), confirm that they had regular access to the Internet, and agree to undertake the TWD for 12 weeks. Participants with any serious medical conditions that would prohibit them from dieting (eg, cancer, bowel disease) were excluded. All eligibility screening was completed online automatically using simple rules/filters. No data were collected regarding the number of ineligible participants. After a brief screening, eligible participants were shown an online information sheet and provided their consent to participate in the study.

Following screening, 8112 people successfully registered to be part of the trial and were randomized to one of the original versions of the TWD portal balanced by age, sex, and BMI. This randomization was achieved through a script designed by our software engineer. Registered participants were queued for condition allocation according to decreasing BMI. The queue was partitioned into 6 buckets that split the users according to gender and age, for which there were 3 buckets. Allocating users to experimental conditions involved processing each bucket in turn and allocating the next available condition number to the next user, thus ensuring that each condition received the correct proportion of users of that age, gender, and BMI.  Only a small number of participants were allocated to the information-based site since one of the major interests of the study was a comparison of interaction with specific website features (Multimedia Appendix 2), and these were minimal for the information-based site.

Participants were blinded as to the condition they were allocated. All were told that CSIRO was evaluating a newly developed online version of the TWD program. Participants were not aware of the features available or the differences between website conditions at any point of the study.

The successful registrants were sent an email thanking them for enrolling in the study and informing them of the projected study start date (approximately 4 weeks later). Due to the unexpected volume of interest in the study, this date was revised by a further 2 weeks and participants were informed via email.

**Figure 1:**
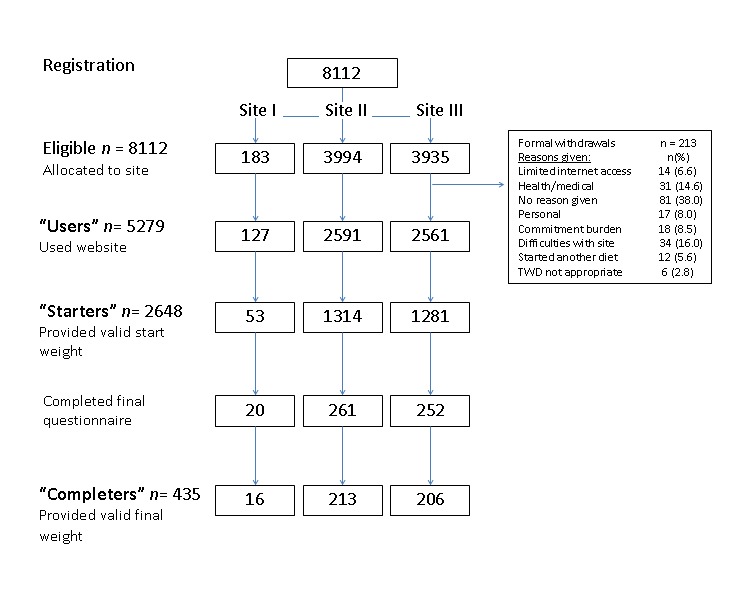
Allocation of participants to websites and numbers remaining at each stage. Site I= Information-based; Site II=Supportive; Site III=Personalized-supportive.

Of those who registered, 65.1% (n = 5279) actually accessed the website when it became available (Figure 1). This group was classified as “Users” of the website. Of the users, 2648 provided a valid baseline weight at the commencement of the 12-week trial (6 weeks after registration) and became our sample for the purposes of the weight loss data (referred to as “Starters”).

Once the trial began, participants had unlimited, free access to the version of the website they were randomized to. Participants randomized to the supportive site conditions could use the discussion forum to contact the study team with any technical or dietary enquiries. All users, including those randomized to the information-based site, could also email the team. During the 12 weeks, 7 group emails were sent from the “The Online TWD Team”, which thanked users for their involvement in the trial, described some group-level data (eg, number of users on the site), and encouraged them to visit the site.

At the end of the 12-week period, participants were further thanked for their participation and asked to complete a follow-up questionnaire and an evaluation survey. As an incentive to complete this survey, participants were offered the chance to win one of 3 vouchers for 150 AUD during week 12 of the study. Just over 5% of those who originally registered provided follow-up weight values; these users are referred to as “Completers” (Figure 1).

### The Online TWD Portal

**Figure 2:**
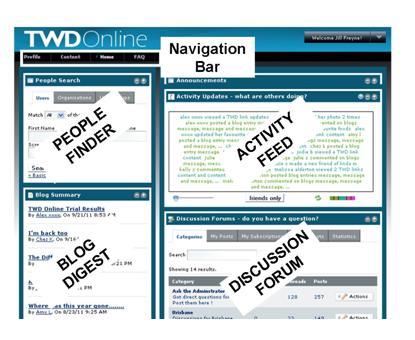
Example of a screenshot from the TWD Online portal.

The Online TWD Portal was built using a customized version of the open source Liferay Portal software [30] running on an Apache Tomcat server and using an Oracle database for relational and non-relational data (see Figure 2). Using Liferay allowed the Online TWD to be assembled from existing and custom social, content management, administration, and other portlets, themes, layouts, and plugin hooks. Portlets such as the meal planner and an activity feed were custom built for the TWD Portal.

Access to the website was restricted to those registered for the study. Usernames and encrypted passwords were required to access the site. Each user account was associated with a unique email address. Cookies were not used to deter the creation of multiple accounts, but usernames and passwords were provided to participants via their email only, thus verifying the validity of the email address. Each feature of the website is described briefly below. Further details and screenshots are viewable in Multimedia Appendix 2.

#### Diet and Exercise Information

The information pages included details on the diet, including 160 recipes, 22 exercises, menu plans and shopping lists, alcohol management recommendations, success stories, quizzes [31], and other health-related links. These pages formed the complete website for users randomized to the information-based site and one component of the supportive website.

Dietary and exercise prescriptions were taken from the TWD commercial books [29,32]. The TWD is a structured, nutritionally balanced eating plan for weight control that was developed on the basis of clinical research surrounding the efficacy of high-protein diets for promoting weight loss [33]. The high-protein diet on which the TWD is based has demonstrated weight losses of 7.6±0.4 kg over 12 weeks under clinical research conditions (including face-to-face sessions with dietitians).

#### Homepage

For those participants randomized to one of the supportive websites, several additional tools and services were provided in addition to the dietary information. Access to each component was achieved through a central homepage. This page was mostly functional, updating individuals on activity on the site and providing easy access to tools and information that they may have required.

#### Diet Tools

For weight self-monitoring, a simple weight tracker was provided. Participants could enter their weight as often as they wished. Graphical feedback on progress was provided in real-time.

As the TWD book incorporates a static meal planner detailing 2 weeks of structured meals, this was provided to all groups in the form of a table under the “Diet Information” section. Those in either of the supportive site conditions received an interactive planner that aimed to assist participants in planning their daily food intake. A simple drag-and-drop functionality allowed meals from the provided recipes as well as user-contributed items to be added and removed from a daily plan. The planner provided real-time feedback on the compliance of a plan to the rules of the TWD diet, through a compliance bar located below the plan. For those in the personalized-supportive site condition, intelligent recommendations were generated through a custom-designed algorithm that combined information about user preferences for certain recipes (assessed via a short survey at the beginning of the study) with previous meal plans to make personalized meal suggestions.

#### Social Support

The Online TWD portal facilitated social support through a social networking platform. Within the social networking system, each participant was represented by a Profile Page, which contained space for a photograph and image gallery, personal details, a message board, and a personal or public blog. Users could change default profile names (firstname.lastname) at any time during the study. Friendships between participants could be requested with requests confirmed or denied as the recipient saw fit (referred to as “Friending”). Access to all content on a profile page was restricted to confirmed “friends”.

In the discussion forum, participants could ask questions, provide support, seek advice, and discuss ideas and thoughts with the community at large. Technical and dietary questions were addressed through the forum by an appropriately qualified member of the research team who did not participate in the subsequent analysis. Summary information pertaining to the social networking activities of friends was presented via a News Feed on the homepages. The list of activities in the feed was hyperlinked, such that each activity linked to the relevant page or component of the site and the profile of the user that performed the activity. Finally, participants could interact with a Social Quiz by voting for those of their friends who met certain descriptions (eg, “Best Blogger”). These quizzes were based on interaction with the site and not weight loss.

#### Website Updates During the Trial

At the launch, technical difficulties were experienced by the site, which took 5 days to resolve fully. Participants were informed about these delays by email and advised when they had been rectified. Usage data were collected after this period. User feedback informed a series of updates to color and text size changes as well as minor reorganization of content links to provide quick access to popular content such as meal plans.

### Study Measures

#### Weight

Weight was self-reported in an online questionnaire at both baseline and 12 weeks. Percentage of baseline weight lost was calculated and used as the primary outcome. Baseline weight was considered as the first weight entered online during week 1 or 2 of the trial and was available only for starters (n = 2648). Final weight was taken from the final questionnaire and was available for completers (n = 435). Although 533 participants completed some aspect of the final questionnaire, they either failed to provide a weight or did not enter a valid weight. Implausible values were determined through a series of screening procedures utilizing scatterplots and outlier analyses.

#### Participant Characteristics

Body dissatisfaction [34], proactive coping (Proactive Coping Scale)[35], Weight-Loss Self-Efficacy [36], perceived need to lose weight (single item), and perceived behavioral control and behavioral intentions to stay on the diet for 12 weeks based on the Theory of Planned Behavior [37] were measured using an electronic questionnaire.

At baseline, further data were collected about participants including sex, age, location of residence, and how they use the Internet [38].

#### Usage

The level of usage of the site was assessed via 3 metrics: the total number of days that the site was used, the day during the study when a user’s final action was performed (ie, the last day the site was used), and the number of days that a user was a “member” of the site (days between first and last use of the site).

All interactions with the Online TWD were digitally logged such that each user action type was recorded in our secure database. Fifty-six potential usage activities were summarized into 16 activity groups. The total number of days on which a user recorded an activity from each of the groups was used to operationalize use of the online features. Thus these potentially ranged from 0-84 days.

#### Evaluation

At the end of the study, participants completed an evaluation survey that asked their attitudes toward the website using questions developed by the authors. These questions included “Overall how much did you like the TWD website?” and “How much did the website help you stick to the TWD?”, which were rated on a 9-point bipolar scale from 0 (not at all) to 9 (completely). Perceived ease of use was asked, “How difficult was it to use the website?”, and rated on a scale from 0 (very challenging) to 9 (not at all challenging).

### Statistical Analysis

Retention data were analyzed for all users, whereas other analyses were based on the sample of starters. These analyses were performed by a statistician who was not involved with data collection and randomization of participants.

A multiple imputation (MI) method using the MICE package [39] in the R statistical package [40] was used to impute missing weight loss values for the purposes of intention-to-treat analyses. Final weight loss values for starters who did not complete were imputed using the initial weight, the weight loss calculated from the last online entered weight (taken from the weight tracker), and the date of the last online weight entry as predictive variables. Predictive mean matching was used for the MI [39]. One hundred datasets with imputed values of final weight were generated, and the results of analyzing each of these were combined using the pooling approach [41], thus allowing for the uncertainty in the imputed values.

Analysis of weight loss data was performed using 3 predictive models for both the intention-to-treat and completers datasets. The first of these models assessed the predictive value of website condition (information-based, personalized or personalized-supportive) and participant characteristics for weight loss. The second analyzed how usage of different features of the website and participant characteristics predicted weight loss. This was performed excluding the information-based site users who did not have access to a majority of features. The third and final model was performed to assess whether feature usage found to be predictive in the second model could be predicted by any participant characteristics. For all models, a predictive model was developed by selecting variables using the Bayesian information criterion (BIC) due to correlations between predictors [42]. All potential predictors for each of the 3 models are presented in Multimedia Appendix 3.

## Results

### Retention of Users

**Figure 3:**
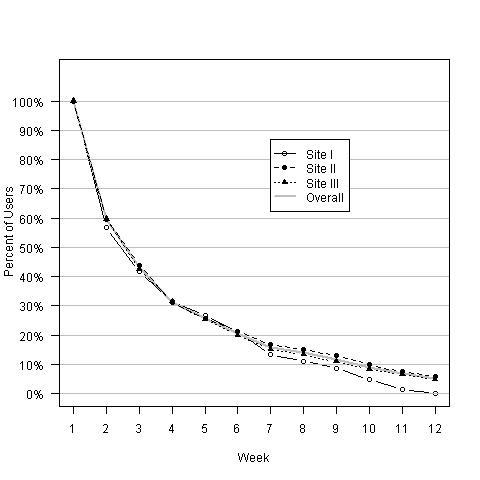
Percentage of users still active in the website throughout the 12 weeks. Site I= Information-based; Site II=Supportive; Site III=Personalized-supportive.

The retention in terms of the proportion of users remaining on the site each week is shown in Figure 3. In the first week of the study, about 40% of users were lost while there was a steady 20%/week attrition of users from week 2 onwards. A total of 5.2% of users had some activity in week 12. The retention each week was compared between sites using a chi-square test. There were no statistically significant differences in retention between the supportive sites (all *P* values >.05). The information-based site had significantly lower retention than the others only at week 12 (*P* = .01).

The median number of days on which a user accessed the site was 2. The maximum was 83 (of a possible 84) resulting in a skewed distribution. Usage difference between websites was statistically significant (*P* = .02) with the average days the site was used at 3.43 (SD 4.28), 5.50 (SD 10.35), and 5.50 (SD 10.35) for the information-based, supportive, and personalized-supportive websites respectively.

### Participant Characteristics for Starters

Demographic characteristics can be seen in Table 2. Most participants resided in the Eastern states of Australia (80.7%), although there were representatives from each of the nation’s states and territories.

**Table 2:** Summary statistics of initial questionnaire measures for starters.

		Non-completer n = 2213 M (SD)	Completer n = 435 M (SD)	Total n = 2648 M (SD)
**Female (%)** ^**a**^		82.1	90.1	83.4
**Age (years)** ^**a**^		44.5 (11.9)	47.4 (11.9)	45.0 (11.9)
**BMI**		34.0 (6.6)	33.6 (6.4)	34.0 (6.5)
	25-29 (%)	30.9	32.4	31.2
	30-40 (%)	52.7	53.1	52.8
	>40 (%)	16.4	14.5	16.0
**PBC** ^**b**^ **(1 min; 7 max)** ^**c**^		6.32 (0.63)	6.33 (0.62)	6.32 (0.63)
**Intention** ^**d**^ **(1 min; 7 max)**		6.75 (0.53)	6.80 (0.47)	6.76 (0.52)
**PCS** ^**a,e**^ **(1 min; 4 max)**		3.06 (0.45)	3.01 (0.44)	3.05 (0.45)
**WLSE** ^**f**^ **(1 min; 4 max)**		2.22 (0.64)	2.24 (0.64)	2.22 (0.64)
**Internet use (%)**				
	<1 to 4 hr	10.8	9.0	10.4
	5 to 10 hr	34.8	33.8	34.6
	11 to 20 hr	24.3	25.3	24.4
	>20 hr	30.2	32.0	30.5

^a^ *P* < .001.

^b^ PBC: Perceived behavioral control over staying on diet.

^c^ *P* < .05.

^d^ Intention: Behavioral intention to stay on diet.

^e^ PCS: Proactive coping scale score.

^f^ WLSE: Weight loss self-efficacy score.

#### Completion Bias

There were differences between starters who did not complete and completers in terms of sex (χ^2^ = 16.86, *P* < .001), age (*t*(2646) = -4.62, *P* < .001), and proactive coping (*t*(2646) = 2.07, *P* = .039), as shown in Table 2. Completers were more likely to be female and were slightly older. Starters who did not complete the study had higher proactive coping than completers, though the effect size was small. Finally, there was a difference in the percentage of completers between the website conditions with starters allocated to the information-based site more likely to complete. Examination of cell counts suggested that a higher proportion of starters who were randomized to the information-based site (30.2%) provided final weights relative to those randomized to the supportive site (16.2%) or the personalized-supportive site (16.1%).

### Usage of Features by Starters

Summary statistics for each of the website usage measures for all starters are given in Table 3. Note that the counts of Blog use and Profile updates were each divided into two different subgroups, since these were found to relate differently to overall retention of users on the site [32]. Blog use was divided into active (contribution) and passive engagement (consumption) with the blog.

**Table 3:** Website features and uptakes for starters (n = 2648).

				Range			IQR^a^	
Features		Description	% using	min	max	Median	Lower	Upper
**General usage** ^b^								
	Day of last action	Last time that an activity was recorded for the user	−	0	87	14	4	39
	Days site used	Number of days that an activity was recorded	−	0	83	3	2	8
	Membership length	Time from the user’s first activity to their last activity	−	0	87	13	2	38
**Diet information** ^b^		View diet or exercise content	99	0	80	3	1	6
**Diet tools** ^c^								
	Weight tracker	Enter an online weight record	100	1	71	1	1	3
	Meal planner	Use the meal planner	68	0	78	1	0	2
	Compliance	View one’s compliance with TWD	36	0	77	0	0	1
**Social support** ^c^								
	View profile: own	View one’s own profile	85	0	82	1	1	3
	View profile: else	View someone else’s profile	30	0	80	0	0	1
	Profile: Text	Add personal information to one’s own profile	30	0	15	0	0	1
	Profile: Image	Add an image to one’s own profile	13	0	22	0	0	0
	Friending	Invite a friend or accept or reject an invitation	14	0	50	0	0	0
	News feed	Follow a feed link	22	0	35	0	0	0
	Blog: View	View a blog	38	0	81	0	0	1
	Blog: Add	Contribute to or comment on a blog	14	0	81	0	0	0
	Discussion forum	Forum posts and comments on others’ posts	49	0	78	0	0	2
	Wall	Contribute to a wall	17	0	78	0	0	0
	Social quiz	Comment on a comparison between two other users	1	0	21	0	0	0

^a^IQR: Interquartile range.

^b^ Includes the information-based site starters (n = 53) who had access only to Diet Information.

^c^ Excludes the information-based site starters.

While there were many features available, many of them were not used. The most used feature was Diet Information, although even for this feature, 75% of starters viewed content on only 6 days of the study period. All starters on supportive sites used the weight tracker, but most used it only a few times. Only 14% established online friendships with another participant.

### Weight Loss

**Figure 4:**
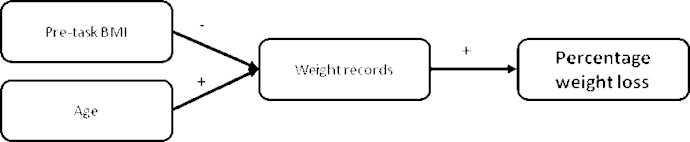
Combined model for variables predicting percentage weight loss.

The distribution of weight loss at the end of 12 weeks is shown in Figure 4, both for completers and using multiple imputed values. The percentage of weight loss from baseline according to MI (n = 2648) was 2.76% (SD 3.56) with 23.7% of starters losing a clinically relevant amount of their baseline weight (>5%). Completers (n = 435) lost 4.10% (4.05) of their initial body weight on average, with 37.6% losing over 5% of their initial weight.

### The Effect of Website Condition and Participant Characteristics on Weight Loss

Average weight losses among completers were 4.15% (SD 4.26), 4.22% (SD 4.34) and 3.97% (SD=3.73) for those who received the information-based, supportive, and personalized-supportive websites respectively.

Results for models predicting percentage weight loss are based on values derived from intention-to-treat analyses using multiple imputation methods rather than completers’ data, as, while specific estimate values differed, significant predictors were the same.

The pooled model fit to predict percent weight loss from the participant characteristics and website condition found no predictors with coefficients significantly different from zero, indicating no differences in weight loss between the 3 groups. The number of predictors selected using variable selection with the BIC criterion in the 100 MI datasets varied from 0 to 4 with no predictor selected in over 50% of the datasets.

### Predicting Weight Loss From Feature Usage

Fitting a model using all of the predictors to the MI datasets gave pooled parameter estimates given in Multimedia Appendix 3. The only predictor that had parameter estimates significantly different from zero was the number of days the weight tracker was used. Between the 100 datasets, the number of predictors selected varied from 1 to 5 using the BIC. Days the weight tracker was used were selected in all of the models while no other predictor was selected in more than half of the models. The average value of *R* ^2^ for the selected models was 10.6.

### Prediction of Site Usage Measures From Participant Characteristics

**Figure 5:**
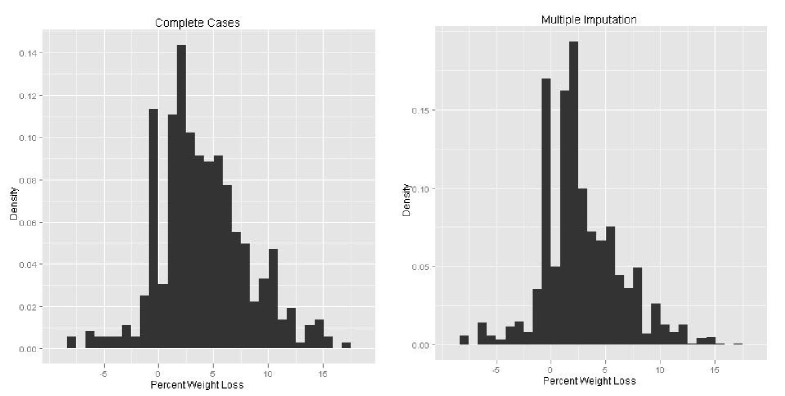
Histogram of percentage weight loss for Completers (left) and estimated using multiple imputation (right).

As usage of the weight tracker was predictive of weight loss, a model predicting use of the weight tracker from participant characteristics was created. Age and initial BMI were selected using the BIC as predictive of uses of the weight tracker (Table 4). Age was a highly significant predictor with older participants providing more days of weight record. Individuals with higher initial BMI recorded their weight less often. Combining the results of both analyses leads to the model depicted in Figure 5.

**Table 4:** Summary of parameter estimates for prediction of percent weight loss and use of the weight tracker (data from the supportive site and III starters only).

		Estimate	*SE*	*t*	*P*
**Weight loss prediction from feature usage and participant characteristics**					
	Constant	1.82	0.42	4.32	<.001
	Weight Tracker	0.34	0.04	7.64	<.001
**Weight tracker prediction by participant characteristics**					
	Constant	1.95	0.41	4.71	<.001
	AGE	0.04	0.01	7.40	<.001
	Starting BMI	-0.03	0.01	-3.26	.001

### Evaluation

Evaluation data were provided by 431 users. There were no significant differences between the supportive sites for any of the evaluation questions. Comparing the information-based site with the pooled results for supportive sites showed significant differences for ratings of liking and the usefulness of the website for supporting the diet, but there was no difference for difficulty of use (Table 5). Regardless of website condition, the most frequent response to “How much did the website help you stick to the TWD?” was “not at all” (a score of 0). While over half of the people in the supportive site (58.4%) and personalized-supportive (57.8%) conditions would recommend the site to a friend, only 27.8% of those in the information-based condition would recommend it (χ^2^ = 6.48, *P* = .039).

**Table 5:** Means, standard deviations, and *P* values for ratings of liking, difficulty to use, and helpfulness of the websites presented by website condition (labelled means without a common letter differ significantly according to Bonferonni-adjusted pairwise comparisons, *P* < .05).

	Information-based n = 18		Supportive n = 209		Personalized-supportive n = 204		
	M	SD	M	SD	M	SD	*P*
Overall how much did you like the TWD website?^a^	2.72^a^	2.24	4.50^b^	2.69	4.36^b^	2.52	.021
How difficult was it to use the website?^b^	4.39^a^	3.31	4.47^a^	2.73	4.44^a^	2.57	.985
How much did the website help you stick to the TWD?^a^	2.39^a^	2.45	4.43^b^	2.91	4.03^a,b^	2.88	.012

^a^ Response format: 0 (not at all) to 9 (completely).

^b^ Response format: 0 (very challenging) to 9 (not at all challenging).

## Discussion

This is one of a limited number of papers to investigate the impact of an interactive/supportive, purely web-based weight loss program. The different levels of support and personalization studied appeared to have little effect on retention, although they were associated with higher average use of the site. We had expected that enhanced functionality, including social support, interactivity features, and/or personalized meal planning, might improve weight loss, but this was not the case for the website evaluated. For those in our sample who received a supportive version of the program, use of the online weight tracker was the only predictor of weight loss.

Rothert et al [23] reported 50% greater percentage of weight loss from baseline for participants who used a tailored/expert versus static system. In contrast, van Genugten et al [24] reported no differences in anthropometric measures between a static and interactive weight management intervention and suggested that “sub-optimal” use of the interactive system may be responsible for their null effects. Unfortunately, Rothert et al [23] did not include objective usage data in their paper and did not report detailed information on their system usage. The more interactive websites in our study were not more effective than the static site in terms of weight loss or weekly retention. It is possible that the level of interactivity provided by the Online TWD was not sufficient to provide any additive effects beyond the diet, which has a high level of structure and which was already available as a commercial book.

Completers in our study lost just over 4% of their initial body weight, on average, with 37% losing a significant amount of body weight (>5%). Other studies of large cohorts of subscribers to commercial weight loss programs have reported slightly higher but comparable weight loss results. Completer analysis of 837 members of the site, vitkklubb.se, indicated an average of 6.1% weight loss at 3 months with close to half of these members reporting clinically significant losses [43]. Evaluation of a cohort of members of the site, biggestloserclub.com.au, was similar with a mean of 6.2% weight loss at 3 months, although fewer members of this site achieved clinically relevant losses (21%) [44]. The average weight loss of around 4% was about half that found in clinical trials of the TWD but reached a larger segment of the population with a considerably smaller investment of resources.

Retention rates are a critical aspect of web-based interventions [45]. Despite the national reach that the Online TWD Portal achieved at the registration stage, retention and uptake of the site were low. Roughly 65% of registrants used the website, which resembles that reported in similar lifestyle interventions [20]. As well as uptake, nonuse attrition is common in web-based studies. Despite up to 21 email reminders, Rothert et al [23] lost 70% of their sample at a 12-week follow-up. At 12 weeks, other purely web-based weight loss programs have reported retention rates of 6% to 35% [43,44]. In contrast, Stevens et al [46] reported that 80% of their sample were still logging into their weight maintenance system after 12 months of intervention. Frequent email and telephone prompts may have contributed to this high retention rate. At the end of our trial period, we retained less than 6% of initial registrants and 16.3% of users who provided a start weight. The Online TWD program included limited prompts, was not commercial, and had no membership fees, which may have reduced the commitment of participants and inflated attrition levels.

Interaction data suggested that many of the features provided in the interactive sites were not heavily utilized. Despite the presence of interactive features, participants’ average scores for liking of the site were low, and this may have reduced their interaction with the portal. Alternatively, participants may not have been experienced with some of the concepts of social networking (such as friending) or hesitant to send requests to people not familiar to them as much of the sample were strangers at commencement of the study (friending was used by only 14% of starters). Relative to previous studies, the uptake of our features may not be as low as they appear. Kelders et al [20] found that under half of their sample accessed their site more than once. The most utilized feature in Binks and van Mierlo’s [22] study was used by only 57.8% of their sample.

Salient questions regarding how to keep people engaged and using a website without relying on high levels of participant contact remain. The inability to translate intention into actual behavior has been well documented in health psychology, and the finding that less than half of overweight/obese people interested in starting the diet actually accessed the site probably reflects this. Providing prescriptive individual goals has shown promising results for the promotion of physical activity [47]. Other behavioral strategies (such as implementation intentions) could be used in future studies to encourage interested participants to translate their initial motivation into action [48]. Likewise, more intelligent system designs may also be able to improve uptake of features and improve user engagement with web-based systems in future trials.

The weight tracker appeared to be a critical feature of our supportive websites, as it was the only feature significantly associated with weight loss. This is consistent with literature on the benefits of self-monitoring for general behavior change including weight loss [49]. In their evaluation of a weight maintenance program, Funk et al [50] also reported that the number of weight entries participants entered was associated with less weight regain. Therefore, future studies are needed to further understand how this feature may have positive effects for weight management. It was interesting that use of the weight tracker was a stronger predictor than overall usage of the website for our trial. This suggests that interaction alone may not be the strongest predictor of weight loss and that the form of this interaction is worth consideration.

There is little debate regarding the need for social supportive web-based programs [8], yet constructing a social and dynamic system remains a challenge. Other papers have reported low uptake of forums and social support and even suggested people prefer to meet face-to-face than virtually [15,18,44]. Yet, results of an analysis of live users (n = 84,828) of a web-based health intervention suggested that “social ties” were a critical feature for user engagement with the site and completion of goals [51]. Our finding that participants using a supportive system visited the site more days than those with the static site offers some support to this observation. Replicating and achieving virtual support, especially without personal contact, remains a challenge for web-based interventions.

Completers were older than people who started without finishing. Age was also predictive of greater use of the weight tracker. Interestingly, similar observations regarding age have been made in previous studies [20,50,52]. The idea that older users have higher engagement with a web-based system seems contrary to statistics indicating that fewer older people access the Internet (37% of 65 or older compared to 96% of those under 55 in Australia in 2010) [53]. Although reach may be limited in older groups in terms of Internet-delivered interventions, older members who do commit to an online program seem to utilize the website more. This may be due to higher free time in this age group who have almost double the minutes of free time per day than those between the ages of 15 and 64 [54]. Consequently, those 65 and older who do access the Internet may be particularly receptive to web-based interventions.

The study we have reported on has strengths and weaknesses that need to be considered in the interpretation of results. Although we used intention-to-treat analysis for our sample of starters, this represented a subgroup of all registrants. The self-selected nature of interaction with the system and provision of a starting weight among users may have biased the results and limited the efficacy of randomization. Attrition also left a small number (n = 16) of completers in the information-based condition. Nonetheless, post-hoc calculations suggested that we had over 90% power to detect a 3% difference in weight loss between this site and the supportive sites.

In order to retain higher ecological validity, we did not require participants to attend our clinic to have objective anthropomorphic measurements taken. This means that our weight loss results are based on self-reported data. Despite careful screening of these data, care needs to be considered in the interpretation of our results.

Unlike previous purely web-based weight loss interventions, the Online TWD was based on a validated diet program that has been proven successful through other delivery mechanisms. Despite their relative marketing successes, few commercial programs are supported by an evidence-base [55]. Thus, although the content of our program was not theoretically derived, it was based on an efficacious weight loss program.

Finally, our intervention sites were prototypes and not built to commercial standards. Among the many factors surrounding Internet intervention, “look and feel” can be important drivers of successful uptake [56]. Although it is virtually impossible to disentangle the specific aspects of the site that led to low average liking scores, piloting usability and look and feel may improve attitudes towards the website in future trials.

Previous reviews have attempted to identify the characteristics of successful eHealth weight loss interventions but have not clearly separated Internet and face-to-face delivered components of the programs. Despite limitations, purely online approaches are essential if the value of such interventions for weight loss is to be understood. Our results suggest that provision of a weight tracker may be a promising web-based feature for weight loss; however, more studies are needed to establish why and how this tool can be utilized to promote greater weight loss.

## Multimedia Appendix 1

Study protocol originally submitted to Research Ethics Board.

## Multimedia Appendix 2

Screen shots of the TWD Online Portal includes examples of homepage, diet and exercise information, diet tools, social support features.

## Multimedia Appendix 3

Pooled parameter estimates for model using all of the predictors to the Multiple Imputation datasets.

## Multimedia Appendix 4

CONSORT eHealth V1.6.1 checklist [57].
