# Supplementary material for: Features Predicting Weight Loss in Overweight or Obese Participants in a Web-Based Intervention: Randomized Trial
Source: J Med Internet Res. 2012 Dec 12;14(6):e173. doi: 10.2196/jmir.2156 (PMC3558051; doi:10.2196/jmir.2156)
Supplement: Supplementary file 1 [file jmir_v14i6e173_app1.pdf]

HREC number: **10/02**

Study Title: **Online Total Wellbeing Diet Experience**

[Deviations from the original protocol are noted in orange. These have been submitted as part of our final report to the HREC]

STUDY OBJECTIVE

The overall aim of the project is to investigate the role ICT features in self-reported diet compliance and weight loss.

Goals:

- Develop a prototype TWD portal with varying levels of ICT features.
- Evaluate the relative effect of varying levels of ICT features on diet compliance i.e. Explore how different ICT features of the TWD Portal affect retention, attrition, lifestyle change including self-reported weight loss, self-efficacy, and body dissatisfaction on the TWD program following a 12-week period of interaction.
- Evaluate the presence of and usage of intelligent ICT features designed to increase the retention and overall usage of the diet compliance web site.

System Overview:

The online TWD Portal is an online social diet compliance system aimed at providing users with the skills and information and easy to use tools to assist them with diet compliance

The online TWD Portal has a static content-based component and a dynamic social networking component and diet compliance specific tools. Some of these tools have a research focus and others do not.

The content-based component of the online TWD Portal consists of scientifically validated information on diet and lifestyle primarily taken from the CSIRO's Total Well-being Diet (TWD) book. The content covers information on the TWD diet, 160 recipes, exercises, menu plans, shopping lists, alcohol management, success stories, quizzes, and other health-related links provided in form of RSS ("Really Simple Syndication"). The content is presented in a user-friendly way and the participants were provided with several options to navigate their way through the content.

The main page of the system is the system dashboard. This is the key area of the system and allows users to

- Plan their food intake
- Report on actual food intake
- View activity feeds
- See how they are progressing through visualisations
- Access the TWD content
- Access the social networking and forum components of the system

Summaries of activity information, provided through activity feeds include information pertaining to "friending", commenting, blogging, completing quizzes and forum input as well as all the interactions with the underlying TWD diet content. Activity feeds are therefore one of the core mechanisms that the system uses in order to link the content and the social components to enable complementary and seamless user interaction with the two components.

The social networking component consists of two core areas: a Profile page, and a Forum.

The Profile page provides members with an environment where they can create their online identity. They can represent themselves to others in the form of a profile photograph and personal details. They can express themselves through a blog editor. They can use an activity diary to report their food intake and physical activities performed.

The Forum page provides users with a platform for social support from community members on which they can discuss/share information. It was the intention of the forum that the discussions should centre on healthy lifestyle and that users could ask questions, provide support, seek advice and discuss different ideas and thoughts with the community at large. The forum too is therefore useful in linking the social component and the healthy living content. We seeded the forum with sample relevant threads to encourage user participation in the discussions. The forum is monitored by a system administrator and by domain experts who answer specific health, exercise and nutrition questions posted by participants.

STUDY DESIGN

This study will utilise a longitudinal, repeated measures design with seven experimental conditions. Each condition will require a slightly differing version of the system (V1-V7). Usage of these versions will allow for the ICT impact on diet compliance to be achieved.

V1: No frills. This is basically a static online version of the TWD book with no ICT features. The planner is replica of the plan provided in the book. No central dashboard is provided, no planning or reporting functionality exist, no social network is available.

The features that will be provided are:

- Online static version of the TWD book
- Static menu planner (as in the TWD book)

V2: Standard Features + System-Generated Social Comparison. This is a fully featured system, as described in the system overview above.

Here are the features that will be provided:

- Online interactive version of the TWD book
- Non-personalised interactive menu planner
- Non-personalised activity feeds
- Text-based compliance tracking
- System generated social comparison **[Referred to as “Social Quiz” in manuscript]**
- Social networking features

V3: Personalised Features + User-Generated & System-Generated Social Comparison. This version of the software will include intelligent ICT features. The inclusion of this group allows for reporting on the ICT specific. Here are the features that will be provided:

- Online interactive version of the TWD book
- Personalised interactive menu planner
- Personalised activity feeds

- Text-based compliance tracking
- User-generated & system generated social comparison
- Social networking features

V4: Personalised + visual compliance + User-Generated & System-Generated Social Comparison. This version of the software is very similar to V3 with the addition of visualisation components. Again inclusion of this group allows for reporting on the ICT specific questions. Here are the features that will be provided:

- Online interactive version of the TWD book
- Personalised interactive menu planner
- Personalised activity feeds
- Visualisation-based compliance tracking
- User-generated & system generated social comparison
- Social networking features

V5: Standard Features + System-Generated Social Comparison + Personalized Meal Planner*.* This is a fully featured system, as described in the system overview above.

Here are the features that will be provided:

- Online interactive version of the TWD book
- Personalised interactive menu planner
- Non-personalised activity feeds
- Text-based compliance tracking
- System generated social comparison
- Social networking features

V6: Standard Features + System-Generated Social Comparison + Personalized Feeds*.* This is a fully featured system, as described in the system overview above.

Here are the features that will be provided:

- Online interactive version of the TWD book
- Non-personalised interactive menu planner
- Personalised activity feeds
- Text-based compliance tracking
- System generated social comparison
- Social networking features

V7: Standard Features + Personalized Social Comparison*.* This is a fully featured system, as described in the system overview above.

Here are the features that will be provided:

- Online interactive version of the TWD book
- Non-personalised interactive menu planner
- Non-personalised activity feeds

- Text-based compliance tracking
- Personalized social comparison
- Social networking features

For each version of the system above we require an experimental condition (required user numbers reported later). Each user will see only one version during their 12 weeks.

[Collation of seven versions into three is indicated in the first column of the table. This was done based on core features as several conditions were included with the purpose of answering ICT-specific questions.]

	Core version	Diet exercise info	Meal Planner	Compliance feedback	Social support system	Diet & wt self-monitor	Personalised planning
V1	IB	]	✘	✘	✘	✘	✘
V2	Sup	]	]	]	]	]	✘
V3	Sup	]	]	]	]	]	✘
V4	Sup	]	]	]	]	]	✘
V5	PS	]	]	]	]	]	]
V6	PS	]	]	]	]	]	]
V7	PS	]	]	]	]	]	]

IB= Information-based; Sup=Supportive; PS=Personalised Supportive

SAMPLE SIZE

There are four experimental conditions in total, (V1-V4 from above).

[These were combined into three core conditions after preliminary data screening].

[After consulting with a statistician we amended our analysis plan. Post-hoc calculations suggested that we had over 90% power to detect a 3% difference in weight loss between this site and the supportive sites with the new analyses].

We would like to determine whether there are statistically significant differences in engagement, diet compliance, self-efficacy, self-esteem and body dissatisfaction between the four conditions. A one-way ANOVA would require 45 participants per condition if we set the power calculation to a medium population effect size at power = .80 for α = .05.

In each of the following conditions, there are seven ICT features that could individually or collectively affect engagement: V2-V7. The seven features are: planner, activity feeds, compliance progress, social comparison, forums, “friending”, and content browsing. We would in this case rely on regression analysis to determine the effect of a specific independent variable (or set of independent variables) on engagement, diet compliance, and self-efficacy. A multiple regression analysis would require 103 participants per condition if we set the power calculation to a medium population effect size at power = .80 for α = .05. All the power calculations have been done using G*Power 3 statistical software.

We will therefore need a minimum of 45 participants in V1, and 103 participants in each of the other six conditions. The study therefore requires a minimum of 663 participants (i.e. 45 + [103x6]).

With a projected drop-out of 75% over the 12-week study period, we will contact 2652 people.

RANDOMISATION

Participants will be randomly assigned to one of the experimental conditions. This will be done through a randomisation application which assigns eligible participants to certain conditions. Participants who contact the researchers through snowball recruitment methods will not be randomised into different conditions. These participants will be placed in the same condition as the ‘friend’ who recommended that they contact the study team to avoid un-blinding participants to the features of the different conditions. We will be collecting demographic data in order to test and/or control for any potential effects of demographic similarities. If people in existing friendship networks are very similar, we will use friendship group as a clustering variable to statistically minimise the effects of strong similarities.

RECRUITMENT STRATEGIES

We plan to recruit participants using methods such as:

- Media release
- CSIRO websites and email lists
- Referrals from other participants

In all the recruitment methods, we will use a distributed call for participation which will point potential participants to a website specifically designed to screen participants for our study. We will distribute our media release/news story to the main national and local newspapers in Victoria, New South Wales, Tasmania and Queensland. This is intended to widen our coverage. We will place an advertisement in the following free websites: Gumtree and Craigslist.

[After our initial national media publicity, we no longer needed to rely on other recruitment methods as this attracted a stronger response than anticipated]

In order to maximise our recruitment we will ask those who respond to our call for participation to invite their friends and others to participate to encourage snowball recruitment. Participants will be allowed to inform other potential participants using a customisable email template with the call for participation and the details of the referrer included. People who receive and follow-up the recommendation will be placed in the same condition as their friend.

INCLUSION/EXCLUSION CRITERIA

Eligible participants must:

- want to participate in the TWD program
- be over 18 years of age
- have an Body Mass Index of 25kg/m^2 or above

OUTCOME MEASURES

Primary outcome

Engagement

The primary outcome measure is user engagement with the portal. Engagement is measured on several dimensions which focus on interaction with the system. These include:

- number of days active
- number of weeks active
- amount of time spent on the site
- number of contributions to the site
- duration of active membership

The dimensions will be assessed separately.

Secondary outcomes

Compliance to TWD

Dietary compliance, which will be assessed through 3 separate measures: self-reported dietary compliance, self-reported weight loss, and use of the menu planner.

Psychological constructs

Psychological outcomes including self-efficacy, self-esteem and body dissatisfaction will be assessed. Self-efficacy will be measured using the 20-item Weight Efficacy Life-Style Questionnaire (WEL). This questionnaire has been validated in previous studies [Clark et al., 1991]. It measures how people feel that they can control their eating in five different situations (negative emotions, availability, social pressure, physical discomfort, positive activities). It can be used to assess these dimensions separately or as a global measure of weight self-efficacy.

The Theory of Planned Behaviour [Ajzen, 1985] uses perceived behavioural control to measure self-efficacy. This outcome has been found to predict behavioural intentions as well as behaviour change. It is therefore a potentially important precursor to successful behaviour change.

Self-esteem will be assessed using the 10-item Rosenberg Self-Esteem Scale [Rosenberg, 1965]. This scale measures global self-esteem.

[Self-esteem was replaced with the Proactive Coping Scale]

To measure self-esteem relating specifically to body weight, an adapted version of the Body Dissatisfaction Scale (a subscale of the Eating Disorders Inventory; [Garner, Olmstead & Polivy, 1983]). This asks participants to rate their body shape relevant to its current status and their ideal body shape.

Interaction with portal

Finally, the impact of individual features on the stickiness of the portal will be assessed through examination of interactions logs (regression analysis) as well as through a post-task questionnaire. The Technology Acceptance Model [Davis, 1989] is widely used to evaluate new technologies in the discipline of Information Systems. Questions in the post-task questionnaire on the perceived ease of use have been adapted from using this model. Other questions are designed to evaluate the experience on the TWD program and the features of the website.

[Due to space constraints, we have reported limited evaluation data in manuscript]

PROCEDURE

The potential participants recruited through the above methods will go through an online screening process to participate in our study. The online screening website will provide:

- A homepage providing a brief description of what the study is about – (i.e., the call for participation). This includes a description of the conditions that an individual has to meet in order to participate in the study, the duration of the study, and the computer/internet requirements.
- A short screening questionnaire to evaluate whether they are eligible or not. This includes questions about their computer/internet equipment, willingness to participate in the TWD program, and whether they are over 18.

Once the screening questionnaire has been completed and submitted, volunteers will know immediately whether they are eligible to participate. Non-eligible volunteers will be thanked for their interest. Eligible volunteers will be asked to provide their contact details (e.g. postal address, phone number and e-mail address). They will be sent the detailed information about the study through an information sheet.

Once the initial registration period is complete, the user will be free to interact with the system as often as they please. The system will e-mail participants every five days over the period of 12 weeks providing activity updates relating to the actions taking place on the site.

[We actually only contacted volunteers 7 times over 12 weeks]

At the end of the 12-week study trial period, they will be contacted via email and the TWD portal, and asked to complete the post-task questionnaires.

REFERENCES

Ajzen, I. (1985). From intentions to actions: A theory of planned behavior. In J. Kuhl & J. Beckman (Eds.), Action control: From cognition to behavior (pp. 11-39). Heidelberg, Germany: Springer.

Francis, J., Eccles, M., Johnston, M., Walker, A., Grimshaw, J., Foy, R., Kaner, E., Smith, L. & Bonetti, D. (2004). Constructing questionnaires based on the Theory of Planned Behaviour. Newcastle, UK: Centre for Health Services Research.

Rosenberg, M. (1965). Society and adolescent child. Princeton, NJ; Princeton University Press.

Garner, D., Olmstead, M., & Polivy, J. (1983). Development and validation of a multidimensional eating disorder inventory for anorexia nervosa and bulimia. International Journal of Eating Disorders, 2, 15-34.

Clark, M., Abrams, D., Niaura, R., Eaton, C. & Rossi, J. (1991). Self-efficacy in weight management. Journal of Consulting and Clinical Psychology, 59(5), 739-744.

Davis, F. (1989). Perceived usefulness, ease of use, and user acceptance of Information technology. MIS Quarterly, 13(3), 319-339.
