# Supplementary material for: Features Predicting Weight Loss in Overweight or Obese Participants in a Web-Based Intervention: Randomized Trial
Source: J Med Internet Res. 2012 Dec 12;14(6):e173. doi: 10.2196/jmir.2156 (PMC3558051; doi:10.2196/jmir.2156)
Supplement: Supplementary file 2 [file jmir_v14i6e173_app2.pdf]

TWD Online Portal – Home Page

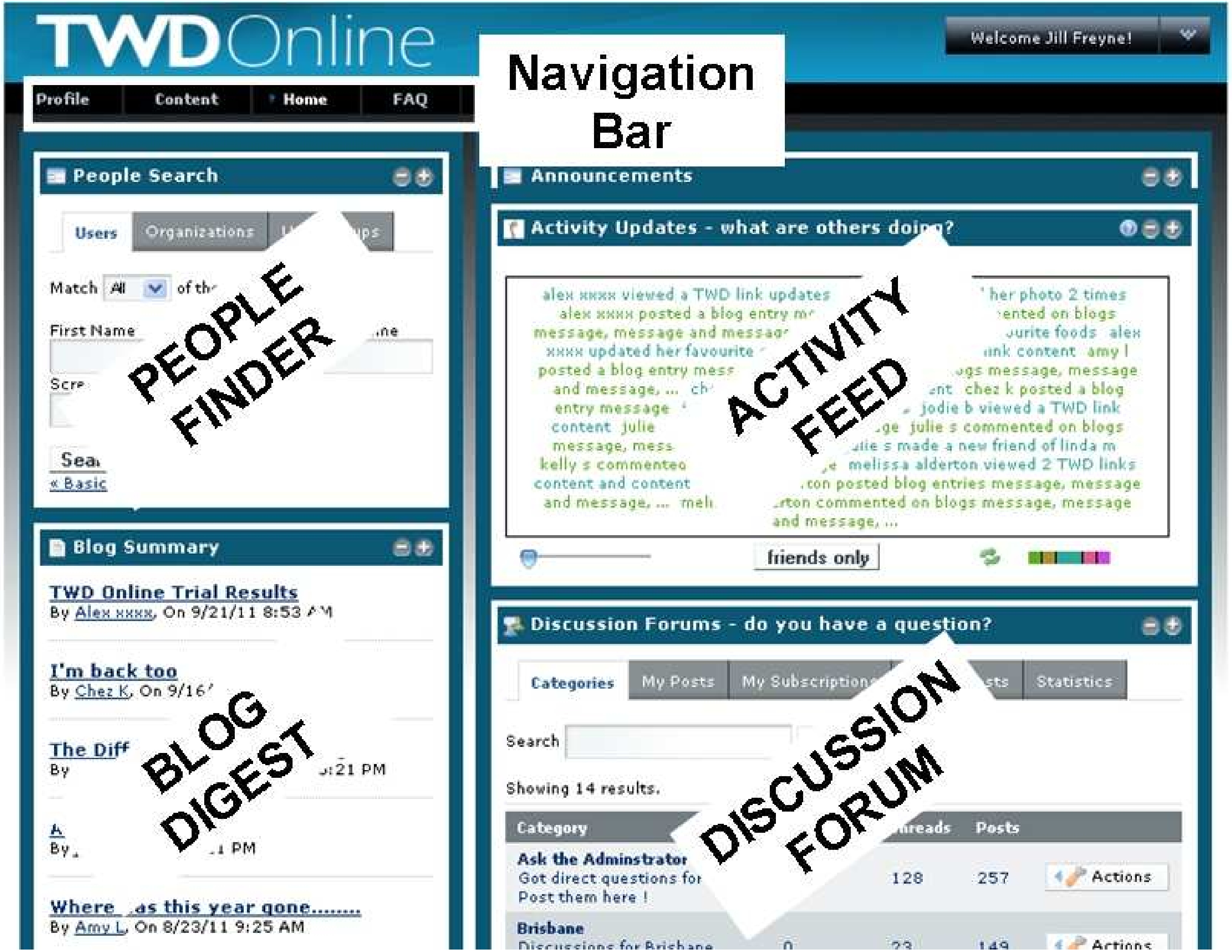

Diet & Exercise Information

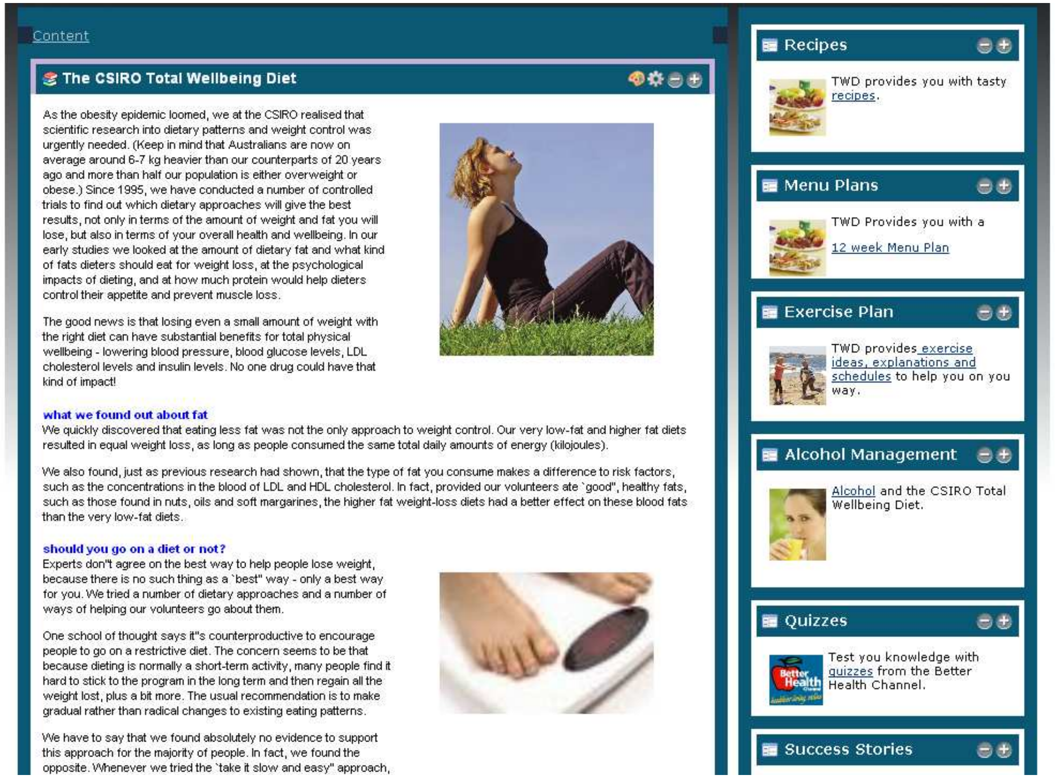

Content

The CSIRO Total Wellbeing Diet

As the obesity epidemic loomed, we at the CSIRO realised that scientific research into dietary patterns and weight control was urgently needed. (Keep in mind that Australians are now on average around 6-7 kg heavier than our counterparts of 20 years ago and more than half our population is either overweight or obese.) Since 1995, we have conducted a number of controlled trials to find out which dietary approaches will give the best results, not only in terms of the amount of weight and fat you will lose, but also in terms of your overall health and wellbeing. In our early studies we looked at the amount of dietary fat and what kind of fats dieters should eat for weight loss, at the psychological impacts of dieting, and at how much protein would help dieters control their appetite and prevent muscle loss.

The good news is that losing even a small amount of weight with the right diet can have substantial benefits for total physical wellbeing - lowering blood pressure, blood glucose levels, LDL cholesterol levels and insulin levels. No one drug could have that kind of impact!

what we found out about fat

We quickly discovered that eating less fat was not the only approach to weight control. Our very low-fat and higher fat diets resulted in equal weight loss, as long as people consumed the same total daily amounts of energy (kilojoules).

We also found, just as previous research had shown, that the type of fat you consume makes a difference to risk factors, such as the concentrations in the blood of LDL and HDL cholesterol. In fact, provided our volunteers ate `good", healthy fats, such as those found in nuts, oils and soft margarines, the higher fat weight-loss diets had a better effect on these blood fats than the very low-fat diets.

should you go on a diet or not?

Experts don"t agree on the best way to help people lose weight, because there is no such thing as a `best" way - only a best way for you. We tried a number of dietary approaches and a number of ways of helping our volunteers go about them.

One school of thought says it"s counterproductive to encourage people to go on a restrictive diet. The concern seems to be that because dieting is normally a short-term activity, many people find it hard to stick to the program in the long term and then regain all the weight lost, plus a bit more. The usual recommendation is to make gradual rather than radical changes to existing eating patterns.

We have to say that we found absolutely no evidence to support this approach for the majority of people. In fact, we found the opposite. Whenever we tried the `take it slow and easy" approach,

Recipes

TWD provides you with tasty recipes.

Menu Plans

TWD Provides you with a

12 week Menu Plan

Exercise Plan

TWD provides exercise ideas, explanations and schedules to help you on you way.

Alcohol Management

Alcohol and the CSIRO Total Wellbeing Diet.

Quizzes

Test you knowledge with quizzes from the Better Health Channel.

Success Stories

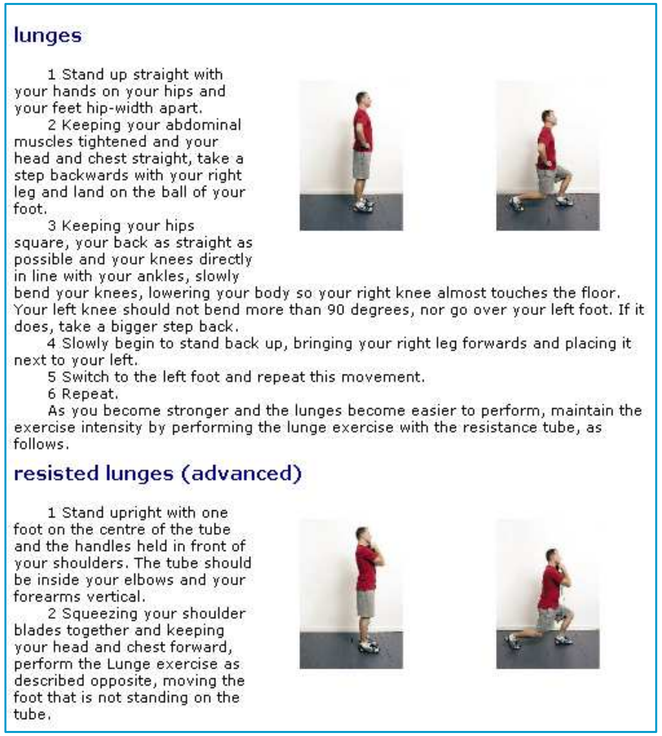

lunges

1 Stand up straight with your hands on your hips and your feet hip-width apart.

2 Keeping your abdominal muscles tightened and your head and chest straight, take a step backwards with your right leg and land on the ball of your foot.

3 Keeping your hips square, your back as straight as possible and your knees directly in line with your ankles, slowly bend your knees, lowering your body so your right knee almost touches the floor. Your left knee should not bend more than 90 degrees, nor go over your left foot. If it does, take a bigger step back.

4 Slowly begin to stand back up, bringing your right leg forwards and placing it next to your left.

5 Switch to the left foot and repeat this movement.

6 Repeat.

As you become stronger and the lunges become easier to perform, maintain the exercise intensity by performing the lunge exercise with the resistance tube, as follows.

resisted lunges (advanced)

1 Stand upright with one foot on the centre of the tube and the handles held in front of your shoulders. The tube should be inside your elbows and your forearms vertical.

2 Squeezing your shoulder blades together and keeping your head and chest forward, perform the Lunge exercise as described opposite, moving the foot that is not standing on the tube.

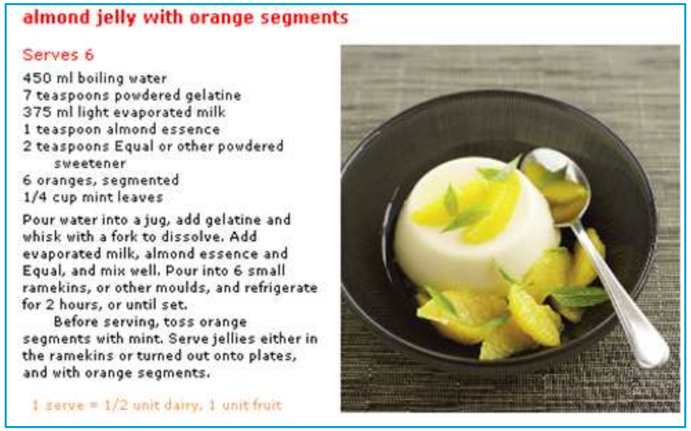

almond jelly with orange segments

Serves 6

450 ml boiling water
7 teaspoons powdered gelatine
375 ml light evaporated milk
1 teaspoon almond essence
2 teaspoons Equal or other powdered sweetener
6 oranges, segmented
1/4 cup mint leaves

Pour water into a jug, add gelatine and whisk with a fork to dissolve. Add evaporated milk, almond essence and Equal, and mix well. Pour into 6 small ramekins, or other moulds, and refrigerate for 2 hours, or until set.

Before serving, toss orange segments with mint. Serve jellies either in the ramekins or turned out onto plates, and with orange segments.

1 serve = 1/2 unit dairy, 1 unit fruit

Diet tools – Weight Tracker

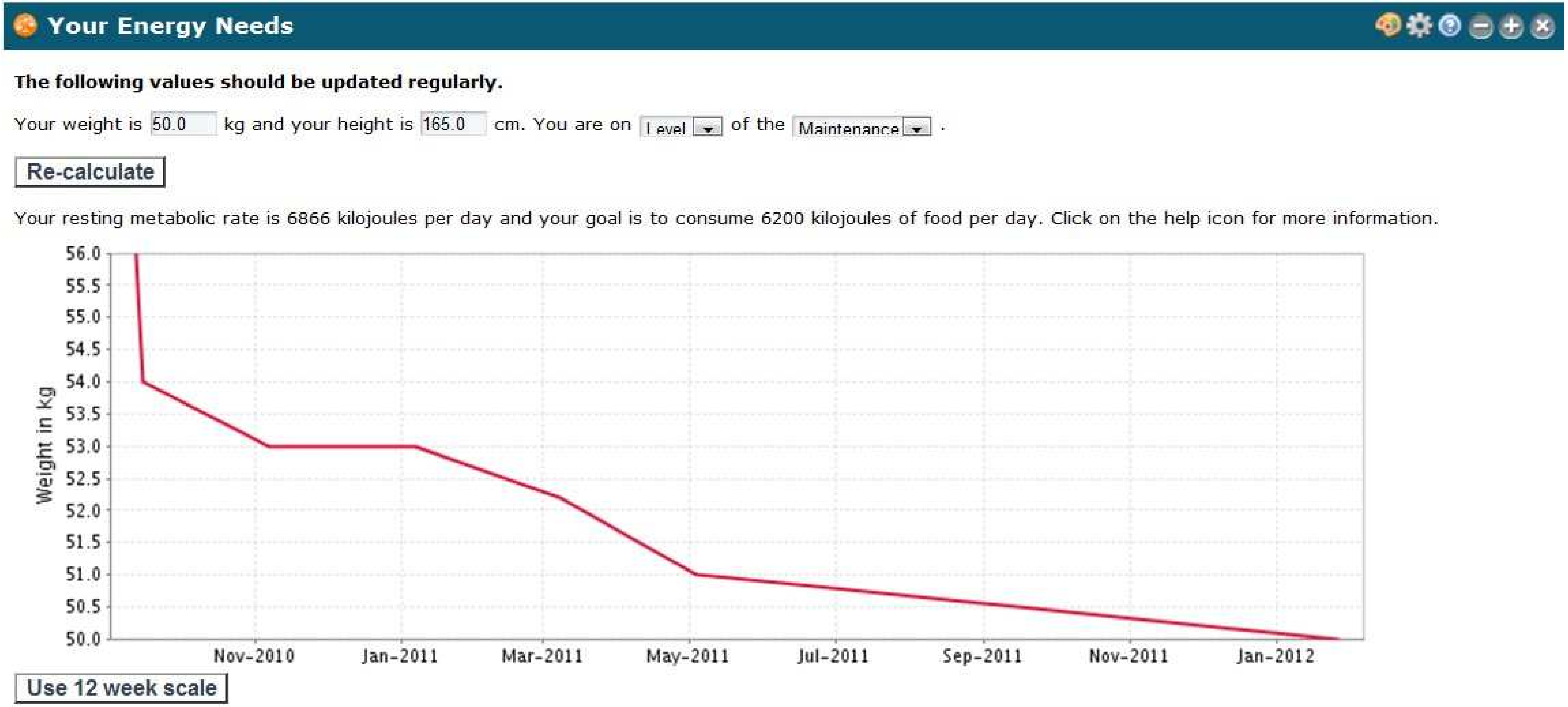

Diet tools – Meal Planner

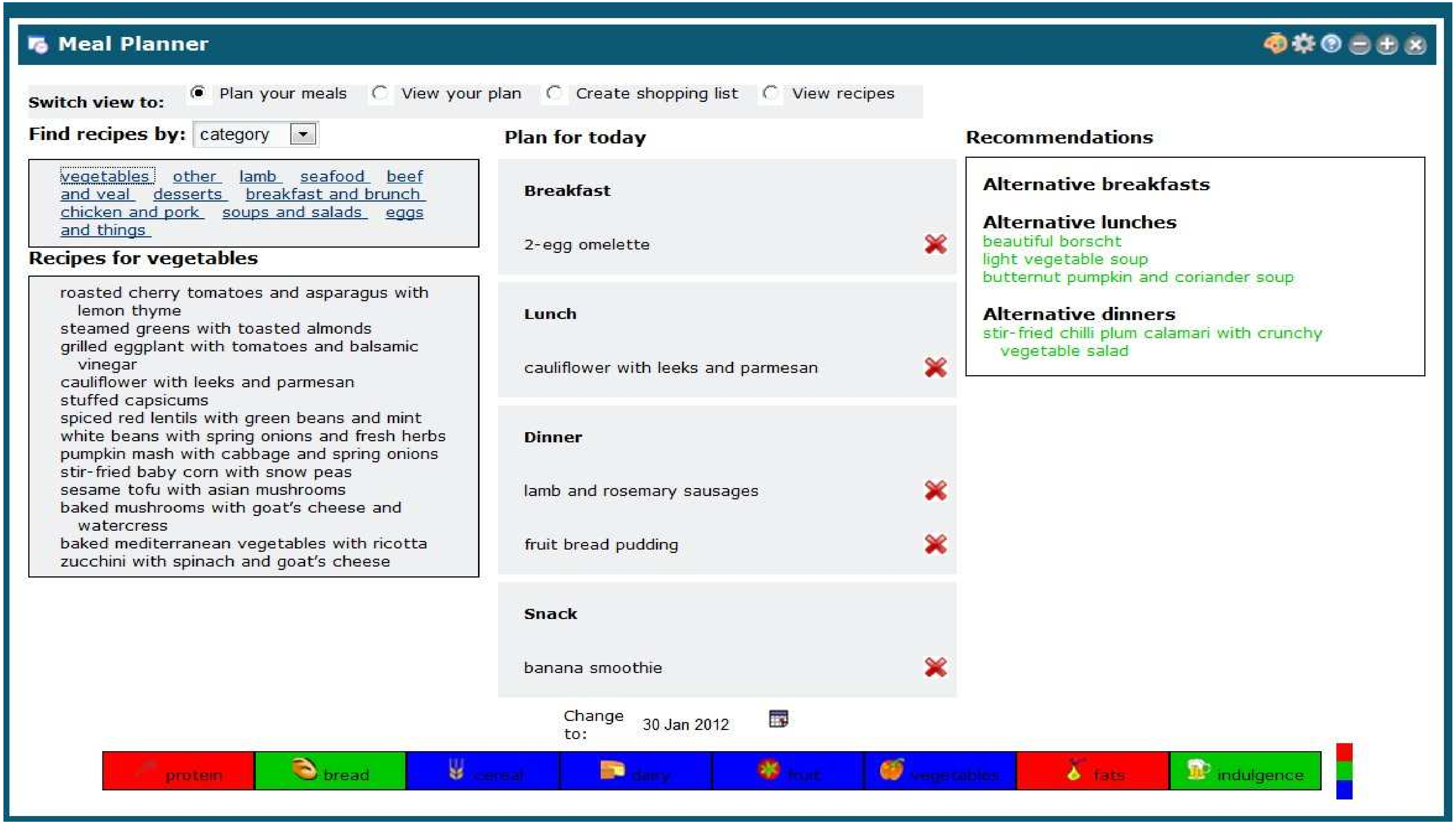

Social Support – Personal Profiles

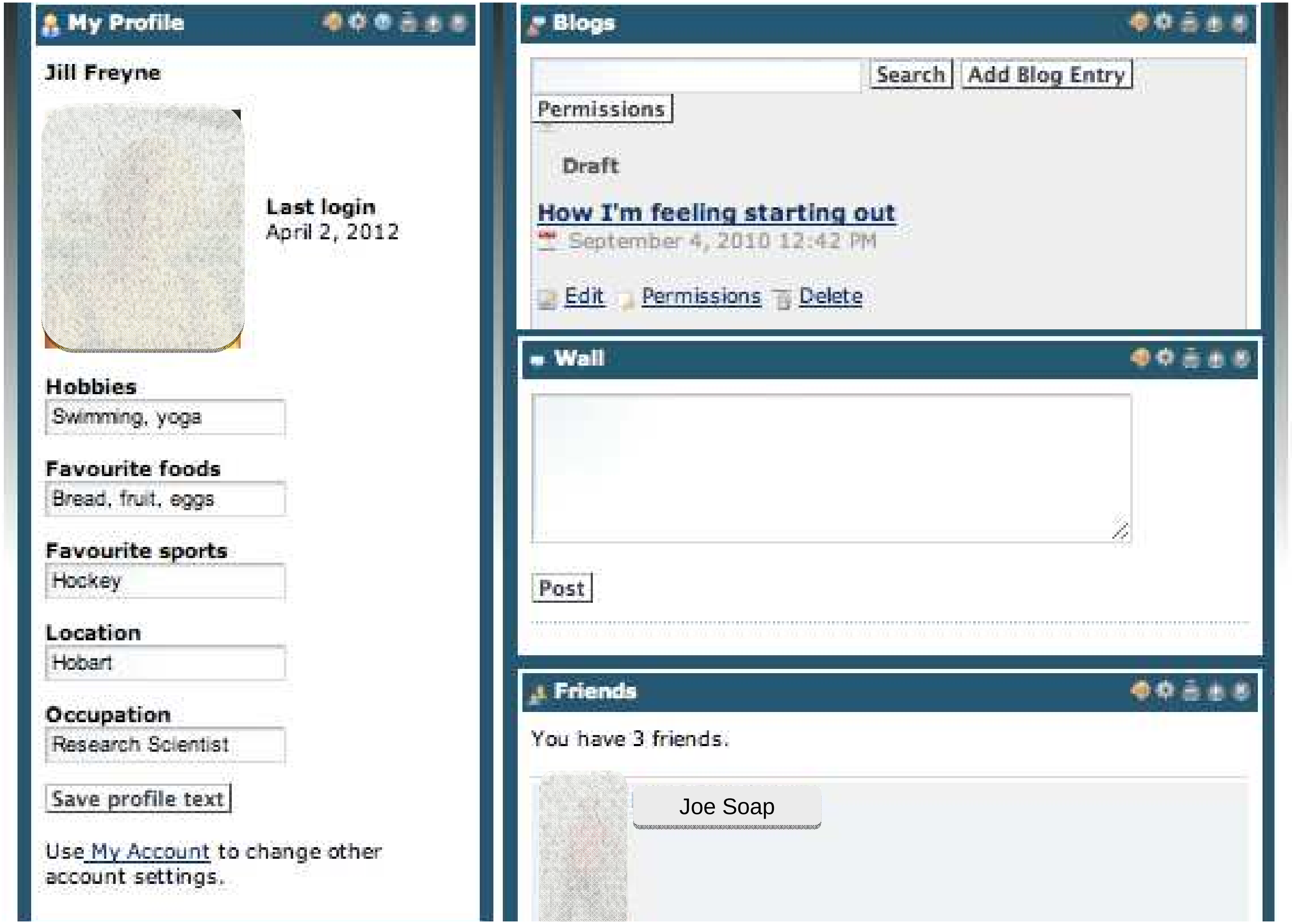

Social Support – Blogs

Blogs

Blogs »

Happy New Year

August 3, 2011 9:47 PM

What? What do you mean it's not New Year's? But on my calender.....what do you mean New Year's is 1st January? On my calender New Year's is 1st August. Of course it is, how could it not be?

She's crazy, I hear you say, she's finally lost it. No, I haven't.

You see it was on 31st July 2010 I started the steps to change my life. Little did I know how such a little decision would have such huge consequences. I never imagined I'd lose 27kgs, I never imagined I'd lose 4 dress sizes, I never imaged I'd lose all the baggage that came with it and become not only fitter and stronger but more confident. I certainly never imagined I'd be running 14kms in the City to Surf.

So, I figure 1st August is as good a time as any to celebrate a New Year.

Therefore, to those of you who started the journey with me -

HAPPY NEW YEAR!

Social Support – Discussion Forum

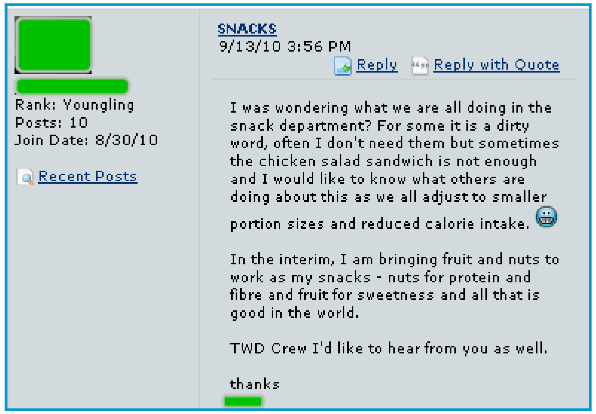

[redacted]
Rank: Youngling
Posts: 10
Join Date: 8/30/10

Recent Posts

SNACKS
9/13/10 3:56 PM
Reply   Reply with Quote

I was wondering what we are all doing in the snack department? For some it is a dirty word, often I don't need them but sometimes the chicken salad sandwich is not enough and I would like to know what others are doing about this as we all adjust to smaller portion sizes and reduced calorie intake.

In the interim, I am bringing fruit and nuts to work as my snacks - nuts for protein and fibre and fruit for sweetness and all that is good in the world.

TWD Crew I'd like to hear from you as well.

thanks
[redacted]

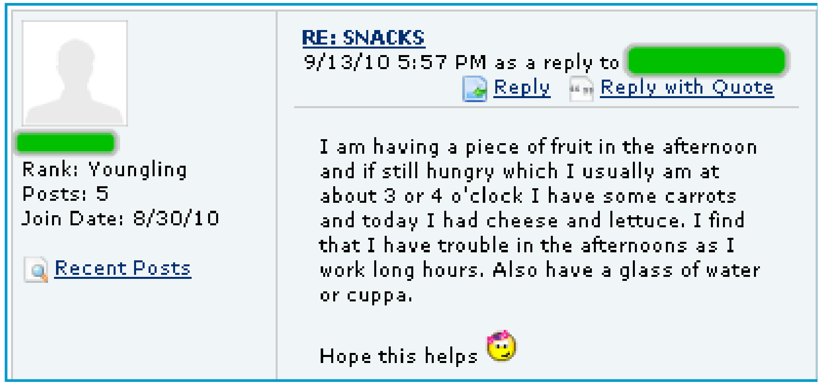

[redacted]
Rank: Youngling
Posts: 5
Join Date: 8/30/10

Recent Posts

RE: SNACKS
9/13/10 5:57 PM as a reply to [redacted]
Reply   Reply with Quote

I am having a piece of fruit in the afternoon and if still hungry which I usually am at about 3 or 4 o'clock I have some carrots and today I had cheese and lettuce. I find that I have trouble in the afternoons as I work long hours. Also have a glass of water or cuppa.

Hope this helps

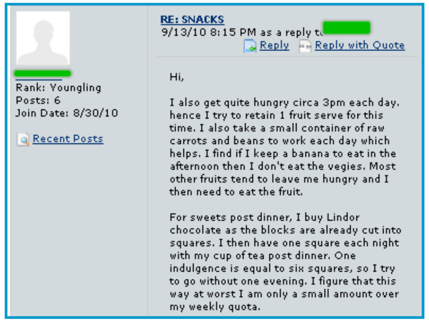

[redacted]
Rank: Youngling
Posts: 6
Join Date: 8/30/10

Recent Posts

RE: SNACKS
9/13/10 8:15 PM as a reply t[redacted]
Reply   Reply with Quote

Hi,

I also get quite hungry circa 3pm each day. hence I try to retain 1 fruit serve for this time. I also take a small container of raw carrots and beans to work each day which helps. I find if I keep a banana to eat in the afternoon then I don't eat the vegies. Most other fruits tend to leave me hungry and I then need to eat the fruit.

For sweets post dinner, I buy Lindor chocolate as the blocks are already cut into squares. I then have one square each night with my cup of tea post dinner. One indulgence is equal to six squares, so I try to go without one evening. I figure that this way at worst I am only a small amount over my weekly quota.

Answers from the community

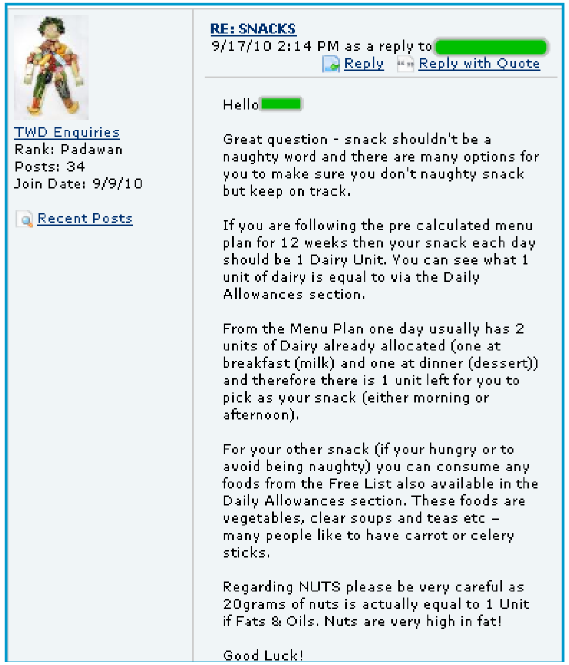

TWD Enquiries
Rank: Padawan
Posts: 34
Join Date: 9/9/10

Recent Posts

RE: SNACKS
9/17/10 2:14 PM as a reply to [redacted]
Reply   Reply with Quote

Hello [redacted]

Great question - snack shouldn't be a naughty word and there are many options for you to make sure you don't naughty snack but keep on track.

If you are following the pre calculated menu plan for 12 weeks then your snack each day should be 1 Dairy Unit. You can see what 1 unit of dairy is equal to via the Daily Allowances section.

From the Menu Plan one day usually has 2 units of Dairy already allocated (one at breakfast (milk) and one at dinner (dessert)) and therefore there is 1 unit left for you to pick as your snack (either morning or afternoon).

For your other snack (if your hungry or to avoid being naughty) you can consume any foods from the Free List also available in the Daily Allowances section. These foods are vegetables, clear soups and teas etc – many people like to have carrot or celery sticks.

Regarding NUTS please be very careful as 20grams of nuts is actually equal to 1 Unit if Fats & Oils. Nuts are very high in fat!

Good Luck!

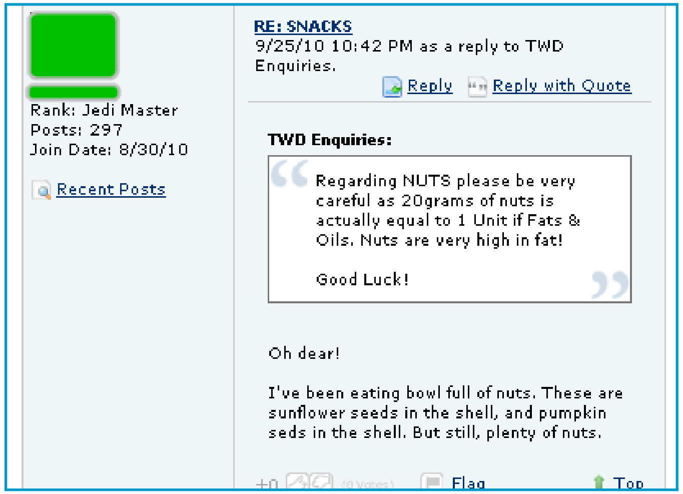

[redacted]
Rank: Jedi Master
Posts: 297
Join Date: 8/30/10

Recent Posts

RE: SNACKS
9/25/10 10:42 PM as a reply to TWD Enquiries.
Reply   Reply with Quote

TWD Enquiries:

> Regarding NUTS please be very careful as 20grams of nuts is actually equal to 1 Unit if Fats & Oils. Nuts are very high in fat!
>
> Good Luck!

Oh dear!

I've been eating bowl full of nuts. These are sunflower seeds in the shell, and pumpkin seds in the shell. But still, plenty of nuts.

+0 (0 Votes)   Flag   Top

Expert answer
