# Supplementary material for: Features Predicting Weight Loss in Overweight or Obese Participants in a Web-Based Intervention: Randomized Trial
Source: J Med Internet Res. 2012 Dec 12;14(6):e173. doi: 10.2196/jmir.2156 (PMC3558051; doi:10.2196/jmir.2156)
Supplement: Supplementary file 3 [file jmir_v14i6e173_app3.pdf]

Multimedia Appendix 3: Pooled parameter estimates for model using all of the predictors to the MI datasets

	Estimate	*SE*	*t*	*df*	*P*
(Intercept)	0.39	2.02	0.20	184.7	0.85
Participant characteristics					
Male	0.28	0.32	0.89	177.3	0.37
Age (years)	<0.00	0.01	-0.25	368.0	0.80
BMI	0.02	0.03	0.74	95.3	0.46
PBC	0.09	0.16	0.53	411.4	0.59
Intention	-0.04	0.18	-0.23	438.6	0.82
PCS	0.1	0.21	0.49	461.4	0.63
WLSE	-0.01	0.14	-0.09	444.7	0.93
Need for wt loss	0.01	0.12	0.11	384.0	0.91
Website (II or III)	-0.07	0.18	-0.37	415.3	0.71
General usage					
Day of last action	<0.00	0.06	-0.07	132.3	0.95
Days site used	<0.00	0.02	0.10	376.0	0.92
Membership length	0.01	0.06	0.22	136.2	0.83
Administrative tasks	0.01	0.04	0.26	530.9	0.80
Diet information	0.04	0.04	0.96	666.5	0.34
Diet tools					
**Weight tracker**	**0.31**	**0.04**	**7.42**	**599.3**	**<.00001**
Meal planner	-0.04	0.04	-0.97	930.0	0.33
Compliance	0.03	0.05	0.58	988.8	0.56
Social Support					
Discussion forum	-0.02	0.03	-0.46	938.2	0.65
View profile: Other	-0.04	0.05	-0.81	1176.0	0.42
View profile: Own	-0.02	0.03	-0.73	1054.4	0.47
Blog: View	-0.03	0.04	-0.79	921.3	0.43
Blog: Add	0.07	0.05	1.36	1305.9	0.17
Profile page: Text	-0.03	0.13	-0.21	508.8	0.84
Profile page: Image	-0.13	0.10	-1.21	1363.8	0.23
Wall	0.01	0.06	0.20	1040.8	0.84
Friending	-0.02	0.07	-0.28	991.8	0.78
Social quiz	0.03	0.14	0.20	2490.8	0.84
News feed	0.07	0.06	1.05	1606.9	0.29

BMI, body mass index; PBC, perceived behavioural control over staying on diet; Intention, Behavioural intention to stay on diet; PCS, Proactive coping scale score; WLSE, Weight loss self-efficacy score
